# Recruitment of 53BP1 Proteins for DNA Repair and Persistence of Repair Clusters Differ for Cell Types as Detected by Single Molecule Localization Microscopy

**DOI:** 10.3390/ijms19123713

**Published:** 2018-11-22

**Authors:** Elizaveta Bobkova, Daniel Depes, Jin-Ho Lee, Lucie Jezkova, Iva Falkova, Eva Pagacova, Olga Kopecna, Mariia Zadneprianetc, Alena Bacikova, Elena Kulikova, Elena Smirnova, Tatiana Bulanova, Alla Boreyko, Evgeny Krasavin, Frederik Wenz, Felix Bestvater, Georg Hildenbrand, Michael Hausmann, Martin Falk

**Affiliations:** 1 Kirchhoff-Institute for Physics, University of Heidelberg, Im Neuenheimer Feld 227, 69120 Heidelberg, Germany; elizaveta.bobkova@outlook.de (E.B.); Jin-Ho.Lee@kip.uni-heidelberg.de (J.-H.L.); hilden@kip.uni-heidelberg.de (G.H.); 2 Czech Academy of Sciences, Institute of Biophysics, v.v.i., Kralovopolska 135, 612 65 Brno, Czech Republic; depesd26@gmail.com (D.D.); ivafalk@ibp.cz (I.F.); evien@centrum.cz (E.P.); olga.kop@centrum.cz (O.K.); bacikovaalena@seznam.cz (A.B.); 3 Joint Institute for Nuclear Research, Joliot-Curie 6, 141980 Dubna, Russia; jezkova.luc@gmail.com (L.J.); marysaveleva@mail.ru (M.Z.); kruglyakovaea@bk.ru (E.Ku.); b-elva@mail.ru (E.S.); bulanova@jinr.ru (T.B.); albor@jinr.ru (A.B.); krasavin@jinr.ru (E.Kr.); 4 Department Radiation Oncology, Universitätsmedizin Mannheim, University of Heidelberg, Theodor- Kutzer-Ufer 3-5, 68159 Mannheim, Germany; Frederik.Wenz@medma.uni-heidelberg.de; 5 German Cancer Research Center (DKFZ), Im Neuenheimer Feld 280, 69120 Heidelberg, Germany; f.bestvater@dkfz.de

**Keywords:** repair foci nano-architecture, ^15^N ion irradiation, single molecule localization microscopy (SMLM), repair cluster formation, repair cluster persistence

## Abstract

DNA double stranded breaks (DSBs) are the most serious type of lesions introduced into chromatin by ionizing radiation. During DSB repair, cells recruit different proteins to the damaged sites in a manner dependent on local chromatin structure, DSB location in the nucleus, and the repair pathway entered. 53BP1 is one of the important players participating in repair pathway decision of the cell. Although many molecular biology details have been investigated, the architecture of 53BP1 repair foci and its development during the post-irradiation time, especially the period of protein recruitment, remains to be elucidated. Super-resolution light microscopy is a powerful new tool to approach such studies in 3D-conserved cell nuclei. Recently, we demonstrated the applicability of single molecule localization microscopy (SMLM) as one of these highly resolving methods for analyses of dynamic repair protein distribution and repair focus internal nano-architecture in intact cell nuclei. In the present study, we focused our investigation on 53BP1 foci in differently radio-resistant cell types, moderately radio-resistant neonatal human dermal fibroblasts (NHDF) and highly radio-resistant U87 glioblastoma cells, exposed to high-LET ^15^N-ion radiation. At given time points up to 24 h post irradiation with doses of 1.3 Gy and 4.0 Gy, the coordinates and spatial distribution of fluorescently tagged 53BP1 molecules was quantitatively evaluated at the resolution of 10–20 nm. Clusters of these tags were determined as sub-units of repair foci according to SMLM parameters. The formation and relaxation of such clusters was studied. The higher dose generated sufficient numbers of DNA breaks to compare the post-irradiation dynamics of 53BP1 during DSB processing for the cell types studied. A perpendicular (90°) irradiation scheme was used with the 4.0 Gy dose to achieve better separation of a relatively high number of particle tracks typically crossing each nucleus. For analyses along ion-tracks, the dose was reduced to 1.3 Gy and applied in combination with a sharp angle irradiation (10° relative to the cell plane). The results reveal a higher ratio of 53BP1 proteins recruited into SMLM defined clusters in fibroblasts as compared to U87 cells. Moreover, the speed of foci and thus cluster formation and relaxation also differed for the cell types. In both NHDF and U87 cells, a certain number of the detected and functionally relevant clusters remained persistent even 24 h post irradiation; however, the number of these clusters again varied for the cell types. Altogether, our findings indicate that repair cluster formation as determined by SMLM and the relaxation (i.e., the remaining 53BP1 tags no longer fulfill the cluster definition) is cell type dependent and may be functionally explained and correlated to cell specific radio-sensitivity. The present study demonstrates that SMLM is a highly appropriate method for investigations of spatiotemporal protein organization in cell nuclei and how it influences the cell decision for a particular repair pathway at a given DSB site.

## 1. Introduction

Ionizing radiation (IR) causes different DNA damages depending on the radiation dose, dose rate, linear energy transfer (LET), photon or particle type, cell radio-sensitivity, DNA repair capacity, etc. [1,2,3]. The most serious damages occur upon high-LET irradiation or high-dose irradiation with low-LET rays, in both cases creating complex double-stranded breaks (DSBs) of the DNA molecule [4]. Such multiple or complex lesions (i.e., DSBs generated in close mutual proximity and often combined with other types of DNA damages) are the most critical for the cell [5] as they highly challenge its repair mechanisms [6,7,8]. Multiple and/or complex DSBs often remain unrepaired and can efficiently cause cell death as successfully used in radiation cancer treatment. On the other hand, in parallel to mediating a high radiobiological efficiency (RBE) of high-LET radiation, the complexity of lesions also increases the risk of mutagenesis, a serious problem, which radiation treatment schemes try to strictly avoid [9,10,11]. These completely diverging aims of radiation therapy highlight the need for research allowing to unequivocally understand the mechanisms of DNA damage and repair.

High-LET, heavy ion radiation, currently represents one of the most potent tools to treat cancer since, in addition to its high RBE, the radiation effectiveness (i.e., the 3D spatial position of the Bragg-peak) can precisely be targeted to the tumor by precise radiation planning and application schemes [12]. Nevertheless, the understanding of DNA damage-inducing mechanisms is important, not only in the context of the treatment and development of diseases, malignant as well as non-malignant (e.g., neurodegenerative). DNA is constantly attacked by environmental factors and repair processes are therefore fundamental biological processes directly related to genome stability, evolution, immune system functioning, and aging. DNA damage is of utmost interest in the field of planned long-term space missions, where exposure of astronauts to mixed fields of ionizing radiation occurring through galactic cosmic rays represents the most serious complication [13]. 

Generation of DSBs in certain regions of the genome leads to specific phosphorylation of histone H2AX in the damage surrounding chromatin, which is manifested as formation of so-called γH2AX foci [14]. Inside these foci, a network of interconnected biochemical pathways, evolved by the cells to counteract permanent DSB injury, operates to remove the lesions and recover DNA integrity. The main pathways are the canonic non-homologous end-joining (NHEJ) [15], the alternative NHEJ [16] and the homologous recombination (HR) [17], which become selectively activated by DNA damage depending on the phase of the cell cycle, chromatin structure at the site of damage, character (e.g., complexity) of DSB and potentially some other factors (reviewed in [18]). Importantly, repair pathways are often deregulated in cancer cells [19], making them defective in DSB repair. Inhibition of the remaining functional pathway is therefore intensively studied as a potentially promising therapeutic approach. On the other hand, despite these defects, some cancer cells remain highly radio-resistant [20]. 

Counting of γH2AX foci as well as foci formed by other repair proteins, e.g., MRE11, 53BP1, RAD51, etc., has become a method well established for intra-cellular dosimetry [21,22,23,24]. For low-LET radiation, a direct correlation between the numbers of DSBs and γH2AX foci has been demonstrated [25]. Nevertheless, it is still under debate how many DSBs are represented by one focus, since staining efficiency, optical resolution of the microscopic instrument used and image segmentation have to be considered and calibrated. On the other hand, upon high-LET irradiation, it becomes obvious that fewer foci can be observed compared to the predicted number of DSBs but the focus sizes are growing with LET [26,27]. This phenomenon can be explained by agglomeration of individual foci. Unexplored internal architecture and complexity of repair foci may therefore largely depend on radiation LET (and local chromatin structure at a given DSB site). In addition, our recent report [1] has suggested that biological effects of radiation largely depend on LET but are determined in a more complex way than simply by this value. During the joint annual congress of the European Radiation Research Society (ERRS) and the German Society of Biological Radiation Research (GBS) in 2017, questions on the extent of the special structure and topology of foci were intensively discussed. Altogether, our observations and the open scientific questions call for an extensive research on the repair focus nano-structure and its relationship with characteristics of the damaging agents, chromatin architecture, and mechanisms and efficiency of DNA repair.

Current investigations using super-resolution light or electron microscopy revealed that γH2AX foci may be composed of several sub-units either called sub-foci or clusters [28,29,30,31,32,33]. Analyzing the spatial arrangement of γH2AX labeling tags after photon irradiation, the data revealed about four separated sub-units according to our measurements [29]. Among these sub-units, some did not contain other DSB related repair proteins [28]. On the other hand, the formation of sub-units was found also for foci of other repair proteins [34,35,36]. The number of repair-focused sub-units seems to correlate with the damage complexity and their topology may potentially influence the cells’ decision-making for a specific repair mechanism at a given DSB site [5,37,38]. Moreover, the structure of foci and sub-units (often called clusters) determined by super-resolution microscopy parameters shows time-dependent re-organization during repair [28,29,34]. 

Beyond the internal γH2AX foci composition and topology, it has become obvious that the structure and topology of the follow-up recruited repair proteins is of central importance to understand the potential of their spatial organization in repair processes. This has reasoned that 53BP1 foci were analyzed in more detail. 53BP1 is not only involved in NHEJ but also acts as a stabilizing factor during HR [35,39]. Our study [40] as well as the studies of others [35,41] using super-resolution microscopy techniques reveal that 53BP1 foci also showed a diversification into sub-units. The individual γH2AX clusters as determined according to fixed parameters of super-resolution localization microscopy do not always co-localize with corresponding clusters of 53BP1 [40]. γH2AX foci are 1–2 megabase pair structures consisting of phosphorylated histones that are formed around sites of DSB damage. In contrast, 53BP1 is a repair protein that binds to methylated regions of histones where it interacts with other proteins, for instance to promote NHEJ. During such interactions, 53BP1 could be displaced from the primary damaged sites [35,42,43], opening them for instance for access of BRCA1 or CtIP [42]. This different behavior of γH2AX and 53BP1 foci, as shown in [35,40], led us to study 53BP1 foci, their sub-unit formation and their time dependent development after high-LET irradiation and during the following repair time independently of γH2AX.

In the present study, we continued to apply Single Molecule Localization Microscopy (SMLM) as described in detail in several recent studies [34,40,44,45,46,47]. SMLM has been successfully applied to analyze DNA repair and cluster formation of γH2AX molecules, i.e., their labeling molecules, respectively, during a long-term cell culture under folate deficiency [48], time course of γH2AX and MRE11 clusters determined by SMLM during DNA repair after low-LET irradiation [29,34] and also heavy ion irradiation as it follows from our proof-of-principle study [40]. These investigations have shown that foci sub-units are formed in a dose and treatment type specific way and first hints are indicating that the chromatin loci of the damages are correlated by similarities [38]. 

SMLM [49] makes use of the advantage to precisely, i.e., in the order of 10–20 nm, localize each labeled molecule in a coordinate matrix. This means a precision in the order of a single nucleosome. Distance and structure calculations based on these image-free datasets can additionally be encoded in image values and thus dramatically improve the microscopic information. However, it might still be challenging to interpret the new sort of data brought about by this method, since it is based on mathematical analyses of signal coordinate matrices of single molecules instead of traditional imaging and computational image analysis. In continuation of our thus far established view on the nano-architecture of chromatin damage and repair loci, we present results of the analysis of 53BP1 foci and sub-units and show the damage response of two cell types with different radio-resistance. 

## 2. Results

By combined means of standard immunofluorescence confocal microscopy and super-resolution SMLM microscopy, we investigated 53BP1 foci formation, persistence, and structural changes during the post-irradiation (PI) period in differently radio-resistant cell types exposed to high-LET ^15^N ions. Neonatal human dermal fibroblasts (NHDF) were used as a model for lower radio-resistance and U87 glioblastoma cells as a model for higher resistance [50]. The selection of these cell types was driven by fact that even small differences would become detectable between extreme cases, if the repair capabilities and thus the 53BP1 recruitment would correlate to radio-sensitivity. The ability of cells to assemble repair foci may be one of the crucial factors influencing the repair efficiency. Since 53BP1 is recruited to γH2AX foci within the first minutes after irradiation, we initially concentrated our research on the early period PI (starting with 5 min PI), during which faster and more prominent structural changes of foci can be expected. Later time points up to 24 h PI were included to get insights into the kinetics of foci relaxation and removal.

Whereas γH2AX foci describe the shape and size of a chromatin locus with damaged DNA, 53BP1 foci provide information on the formation of foci during the recruitment of proteins for repair. In some cell types, these proteins seem to be abundantly available during the whole cell cycle. In others, they are processed immediately after chromatin damaging and transported to the damaged site. Despite similar labeling and microscopy parameters, 53BP1 foci could be better separated one from another than γH2AX foci (for comparison see [1]). 

Firstly, we evaluated the extent and kinetics of 53BP1 focus formation in the cell types studied. As in recent experiments [40], the cells were exposed to 4 Gy of ^15^N ions in a 90°-geometry (i.e., in the perpendicular direction to the cell monolayer) and the 53BP1 foci were qualitatively analyzed by confocal laser scanning microscopy after specific antibody labeling of the repair proteins. These experimental conditions were used for the following purposes: A higher dose of 4 Gy challenges DSB repair systems so that differences in repair capacity of NHDF and U87 cells could be expected to become more apparent. Because a clear discrimination of a large number of individual particle tracks crossing the nucleus is difficult upon this dose applied in 10°-angle, especially at later time points when the clusters determined by SMLM parameters show some diffusional movement by themselves, we coupled the 4 Gy-experiments with a perpendicular irradiation (90°-geometry).

**Figure 1:**
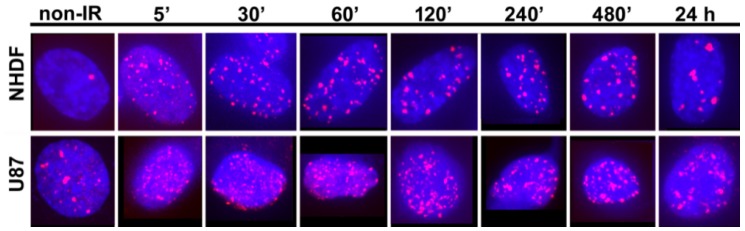
2D maximum intensity projection images of confocal image stacks. Typical examples are shown for fluorescently-labeled 53BP1 foci in NHDF cells and U87 cells after 4 Gy ^15^N-irradiation in 90°-geometry. Along the given time line, samples were taken as aliquots of the same culture at different time points (5 min, 30 min, 60 min, 2 h, 4 h, 8 h and 24 h) after irradiation. All timelines were repeated with independent cultures. For comparison, examples of non-irradiated cells are also presented.

In Figure 1, typical examples of 2D maximum intensity projection images obtained from about 25 confocal slices each are shown for the two cell lines during a time course of up to 24 h PI. Individual images were obtained for aliquots of the same irradiated cell culture fixed at the given time points after irradiation. The series were verified independently by different cell cultures. However, to always start from the same irradiation experiment, each irradiated cell culture was cultivated over more than 24 h and an aliquot was taken and fixed at each time point of repair. In both NHDF and U87 cells, high proportions of 53BP1 foci are visible and the repair proceeded much slower than in samples irradiated with low-LET γ-rays. For further details of foci analysis after heavy ion irradiation in comparison to γ-irradiation, we refer to our recent publication [1]. In NHDF cells showing no or very few 53BP1 signals in non-irradiated samples, compact foci became visible at 5 min PI. The number of foci increased within the first 30 min PI, which was followed by a continuous decrease during the later repair period. However, the foci were still visible at 24 h PI, indicating a persistent repair activity. In contrast to NHDF fibroblasts, U87 cells contained 53BP1 foci also prior to irradiation. In these cells, the recruitment of 53BP1 for DSB repair seemed to be delayed since, up to 30 min PI, the foci appeared less compact and more dispersed than in NHDF cells. The maximum foci formation in U87 occurred at 60 min PI and the number of foci persisting at 24 h PI was significantly higher than that in NHDF fibroblasts. 

Since the formation of sub-units (represented by clusters of labeling points defined by SMLM parameters) within the foci has been described as a topological finding of central importance, the evolution of these sub-units along the particle tracks and their persistence/behavior during the follow-up repair time course was studied by means of SMLM. For these analyses, the cells were irradiated in a sharp (10°) angle to generate the tracks running parallel to the xy-plane of microscopic observation and to achieve the maximum possible resolution and localization precision of labeled molecules in SMLM (10–20 nm). For the same purpose, we also reduced the radiation dose to 1.3 Gy. With this dose, 2–3 particles on average traversed the nucleus, producing well separated 53BP1 protein streaks. Nevertheless, for the reasons already mentioned above, and to obtain SMLM data directly comparable to immunofluorescence confocal microscopy, we also performed quantitative analyses of the formation and relaxation of dense sub-units (clusters) during DSB repair in parallel with the 4 Gy and 90°-irradiation. 

**Figure 2:**
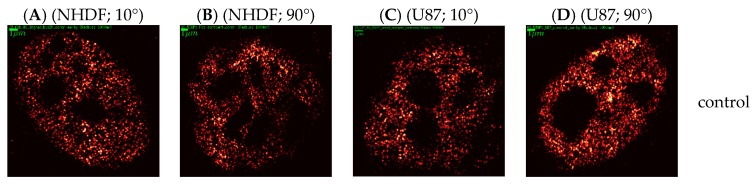

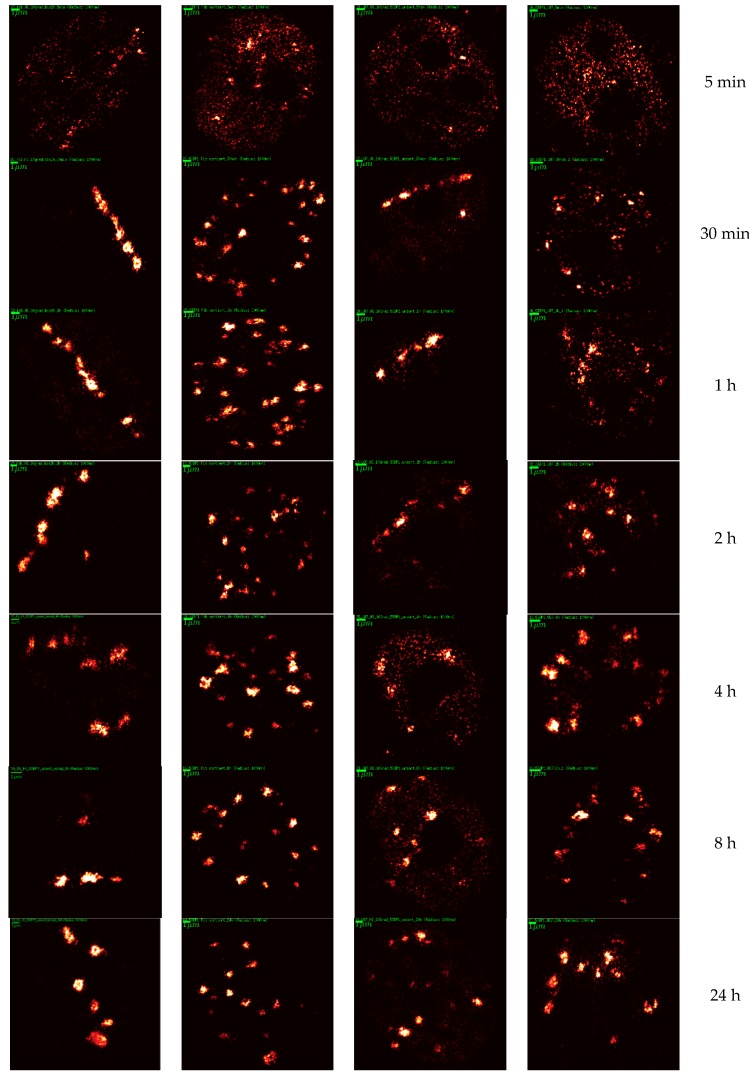
2D density SMLM images of 53BP1 repair proteins. Typical examples are shown for fluorescently-labeled 53BP1 proteins in NHDF cells (**A**,**B**) and U87 cells (**C**,**D**) after 1.3 Gy tangential ^15^N-irradiation (**A**,**C**) (10° angle between the ion beam and the cell layer) and 4 Gy perpendicular ^15^N-irradiation (**B**,**D**) (90° angle between the ion beam and the cell layer). Along this time line, the samples were taken as aliquots of the same culture and fixed at different time points (5 min, 30 min, 1 h, 4 h, 8 h and 24 h) after irradiation. For comparison, examples of non-irradiated control cells are presented. The density images do not show all signal points detected but instead encode the number of neighbors of each signal within a circle of 1 µm radius by the point intensity—the number of signals in the given surroundings around one signal grows as the color of the point changes from red to white. The green bars equal to 1 µm.

In Figure 2, examples of 53BP1 density images are shown for the two cell lines and two radiation schemes inspected at different periods after irradiation. The cell nuclei were obtained from aliquots taken and fixed at different times PI from the same irradiated culture. The density images do not show all signal points detected but instead encode the number of neighbors of each signal within a circle of 1 µm radius by the point intensity. For 10° irradiation scheme, specimens of both cell lines showed characteristic tracks along which the labeling tags were arranged. In the beginning of the DNA repair, individual 53BP1 sub-units of foci lining the track occurred in very close mutual proximity so that their separation by eye was not possible. At later periods PI, the tracks partly dissolved into compact, well distinguished and separately visible protein units. In NHDF fibroblasts but not U87 cells, this separation was accompanied with progressive growth of the focus areas.

According to the results coming from the 90°-irradiation scheme (4 Gy), the number of foci increased until 1 h (NHDF fibroblasts) or even 4 h PI (U87 cells) and afterwards started to decrease continuously, which was, again, as in the 10°-irradiation experiments, associated with growth of the focus areas. The numbers of 53BP1 molecule signals in control (non-irradiated) U87 cells were always higher than in corresponding NHDF samples. On the contrary, during the repair, we always recorded more signals in NHDF cells (up to about 15,000) than in U87 cells (up to about 10,000).

To further investigate the dynamics of 53BP1 molecules, the cluster formation of tagged 53BP1 molecules and their relaxation was studied. Thereby, the definition of clusters (see Section 4.6) followed strict rules of SMLM evaluation, i.e., a minimum number of labeling tags within a given radius. These rules were fixed interactively, as shown for instance in Figure 5. The relative amounts of 53BP1 signal points inside and outside the defined clusters were determined. Let us emphasize that the term “cluster” refers to 53BP1 protein sub-units of microscopically visible foci. Thus, each repair focus is composed of sub-units and these sub-units are quantified as clusters by SMLM parameters. In this context, cluster relaxation refers to a point ensample that no longer follows the limits for clustering. 

**Figure 3:**
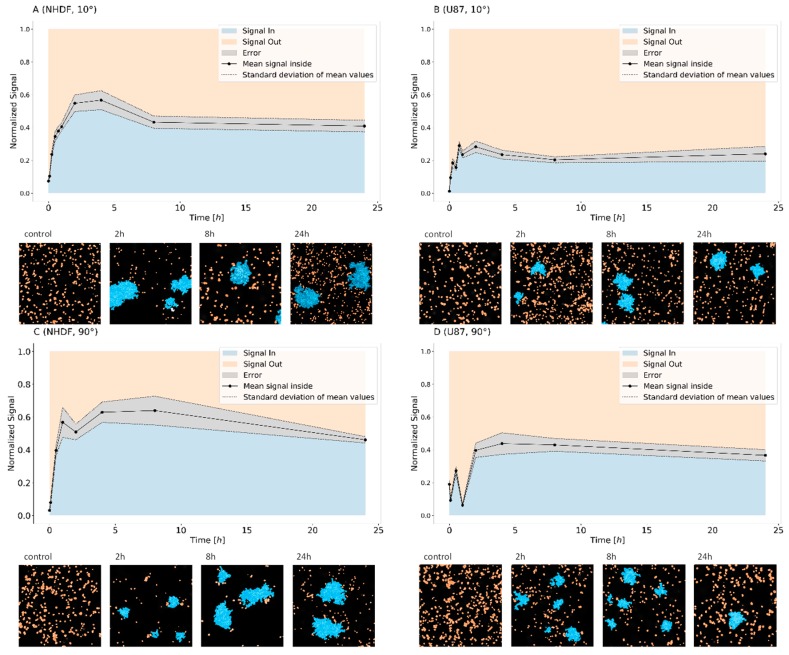
Relative amounts of 53BP1 signals detected within (blue) and outside (orange) repair clusters as defined for SMLM (see Section 4.6). Graphs: Mean values and margins given by the standard deviation are depicted in gray. The values are always normalized to the mean number of signals detected at a given time point. The data are presented for NHDF fibroblasts (**A**,**C**) and U87 cells (**B**,**D**) after 1.3 Gy tangential ^15^N-irradiation (**A**,**B**) (10° angle between the ion beam and the cell layer), and 4 Gy perpendicular ^15^N-irradiation (**C**,**D**) (90° angle between the ion beam and the cell layer). Images: The pointillist images represent examples of sections of cell nuclei with labeling points inside (blue) and outside (orange) clusters at the given time points. The samples were taken as aliquots of the same culture at different time points (from 5 min to 24 h) after irradiation. For comparison, examples of non-irradiated control cells are presented (=0 min).

The clustering dynamics varied for the two cell lines analyzed (Figure 3) but followed the same general tendency for the different doses and irradiation schemes. In NHDF fibroblasts, the average signal number (i.e., number of 53BP1 tags) within clusters rose to more than 60% during the first hour, remained constant until 8 h, and then dropped down slightly at 24 h PI. In U87 cells, the accumulation of signal points in clusters was delayed and not as efficient, although, in non-irradiated cells, a higher basis level of foci existed. An accrual of signals inside clusters occurred not earlier than between 1 and 8 h PI and the average proportion of signals forming the cluster was always considerably lower (up to about 45%) than in NHDF fibroblasts, thereby the more dispersed foci shape was considered by a lower minimum number of neighboring points within a cluster. Based on summarized data obtained for 10°- and 90°-irradiation, we can therefore conclude that normal (non-transformed) NHDF fibroblasts sequester higher proportions of 53BP1 labeling tags within the repair clusters than cancerous and relatively more radio-resistant U87 cells, which leave more than half the amount of 53BP1 outside the foci.

Comparing the 10°- and 90°-irradiation schemes for both cell types reveals a nearly constant mean value of points inside clusters between 8 and 24 h PI for the 10°-geometry, whereas a continuous drop down is observed for 90°-geometry. This may be interpreted by either a cluster relaxation starting from the track border or as a consequence of the different doses. At the higher dose, a couple of clusters may relax after a short time interval since the damage is repaired. Nevertheless, in both cases, a serious number of damages remain unrepaired at 24 h. The latter would stimulate future experiments with longer time intervals PI. 

A long-time persistence of repair foci has often been correlated with the efficiency of DSB repair. Our findings suggest that 24 h after irradiation with 1.3 Gy or 4.0 Gy of ^15^N-ions, the repair processes are still active in both NHDF and U87 cell types. At this period PI, about 14,000–20,000 53BP1 signal points were detected in total in NHDF cells, from which about 40% remained within the defined clusters. However, compared to U87 cells, the relative number of signals in clusters decreased from about 60% to about 40%. Thus, the shape of the curves suggests a rapid repair up-regulation followed by a continuous down-regulation. In contrast for highly radio-resistant U87 cells, about 25% (10°-irradiation scheme) or 35% (90°-irradiation scheme), i.e., 10,000–11,000 signal points, formed these clusters which seem to remain.

In conclusion, the data reveal a long-standing repair activity of 53BP1 protein in both cell types exposed to ^15^N high-LET ions but a different kinetics along the repair period. DSBs generated in the present study by accelerated ^15^N ions are considerably complex but are generated upon a different starting situation. From the foci counting, as in Figure 1, a higher basic level of damaged sites is expected. This is shown by a higher number of 53BP1 foci. Looking on the cluster formation in Figure 3 and assuming that the formation of clusters indicates repair activity, highly radio-resistant U87 cancer cells seem to repair these complex DSBs in a similar way as other damages not induced by radiation, thereby recruiting fewer 53BP1 proteins in clusters than NHDF fibroblasts. 

## 3. Discussion

DNA damage repair is a process controlled by multiple parameters [1,2]. Especially complex damages and DSBs, as could occur after high-LET irradiation, require a diversity of proteins attaching the damaged site in a manner dependent on the repair pathway applied, e.g., HR, canonical NHEJ or alternative NHEJ [5,8,37]. The involved proteins have to address broken ends of the DNA and join them appropriately. Therefore, it seems to be reasonable that the recruitment of repair proteins forms certain sub-units (here called clusters) around the broken ends [28,29,30,31,34,35,40]. In the case of photon-irradiation, several of such clusters, only detectable at nanoscale, contribute to microscopically visible foci [28,29]. They show a typical spatial structure or topology [38] and were found to differ in their repair activity and repair pathway choice [28] indicating the importance of repair foci nano-architecture for the repair mechanisms occurring at given chromatin environments. Important new questions therefore arise about whether the number of these nano-clusters defined by SMLM or rather the number of foci visually separated by confocal microscopy corresponds better to the number of DSBs, and whether the nano-composition of foci influences repair strategy and efficiency at a given DSB site. Not only the spatial arrangement and topology of γH2AX phosphorylation sites may determine the repair process but also the repair proteins recruited to the damaged site appeared in a cluster arrangement, which occurs in an even larger extent after exposure of cells to accelerated particles. In this case, not only components of a single focus but also multiple foci appear to participate in sub-unit or cluster formation, respectively, along the particle track. This explains why the number of foci detected in cells irradiated with different types of high-LET radiation more or less underestimates the real number of DSBs [25,35,40,43].

To better understand the mechanisms behind the DNA damage and repair-induced protein cluster formation and relaxation, we followed the composition of 53BP1 protein clusters/foci during a long post-irradiation period at the nanoscale. By using SMLM super-resolution microscopy [46,47,49], we studied the clusters in two differently radio-sensitive cell types (NHDF and U87) exposed to different doses of accelerated ^15^N ions delivered in two different geometries (10°- and 90°-irradiation). SMLM offered the advantage of the nano-resolution, which was achieved with cells prepared with standard immunofluorescence methods and therefore maintaining their natural 3D-shape [51].

Since 53BP1 is one of the early recruited repair proteins participating in both NHEJ and HR [39,41], it represents an appropriate candidate to study the architecture and dynamics of repair clusters as defined by SMLM and foci composed of several clusters in relation to their importance for DSB repair. Moreover, 53BP1 protein seems to be one of the decision-makers that directs the repair mechanism at a given DSB site either to NHEJ or HR. In contrast to γH2AX, an epigenetic histone modification marking damaged chromatin sites almost immediately after DSB induction, 53BP1 is an early indicator of a starting activity of NHEJ/HR repair machinery. Hence, it is reasonable to investigate the behaviour of 53BP1 during the repair independently. This has been supported by our recent investigation [40] indicating that γH2AX clusters and 53BP1 clusters differ in shape and do not always mutually co-localize.

In the context of the repair pathway chosen by the irradiated cells, the question comes up in which cell cycle phase the cells have been irradiated. Non-synchronized cells were used in this study to better mimic the situation in patients’ tissues during radiotherapy. However, it has been shown [1] that a vast majority of cells was irradiated in a G1 phase of the cell cycle using the experimental conditions described here. The majority of DSBs can therefore be expected to be repaired by NHEJ or the alternative/backup NHEJ pathways. Moreover, recent reports suggest that cells can recover from the cell cycle arrest even in presence of some persisting DSBs. This can happen as soon as the number of DSBs per nucleus decreases to 10–20 [52]. In cell cultures irradiated with low-LET γ-rays, the cells are arrested for relatively short periods, i.e., about 4 h. For high LET radiation, the arrest is significantly longer than for γ-rays, i.e., about 48 h and more [53,54] or even permanent [55], depending on the LET of the ion-radiation. Thus, for the LET of ^15^N as used in the experiments presented here, a checkpoint arrest release can be expected if about 10–15 DSBs remain unrepaired [56]. Thus, the mechanism of repair may be expected to depend more on the location and number of DSBs and the chromatin architecture at the particular damage site than on the cell cycle progress.

In the present study, by means of SMLM, we explored the time course of 53BP1 cluster formation, relaxation, persistence and focus composition on the single molecule level. We found that the accumulation of the labeled protein inside as well as outside the clusters depends on the cell type. This principle behavior seemed to be less influenced by radiation dose or perspective of the particle track (irradiation geometry). For the radio-resistant U87 cancer cell type [50], the relative number of signals in clusters was always considerably lower than the relative amount outside the clusters. This relation does not reflect the absolute number of 53BP1 proteins available but gives information about the “just-in-time” availability. High nucleoplasmic levels of freely floating 53BP1 proteins may point to a permanent repair activity also in non-irradiated cells. This is well compatible with genetically unstable status of cancer cells and presence of an increased (compared to NHDF fibroblasts) average number of γH2AX/53BP1 foci observed here also in cells prior to irradiation. The kinetics seems to indicate rather the recruitment of the existing proteins floating through the cell nucleus than a de novo production and directed recruitment to the damaged site. In contrast to U87 cells, normal non-transformed NHDF fibroblasts showed no or very little repair activity in non-irradiated cells and recruit the repair proteins just-in-time for the repair cluster formation. Nuclear levels and distribution of repair proteins prior to DNA damage induction could therefore be potentially causatively linked to the cell-type specific radio-resistance, at least partially and/or in some cell types. Our results also support the idea that different cell types may vary in the preferred mechanism of DSB repair and, thus, requirements for particular repair proteins including 53BP1.

A lower co-localization of γH2AX and 53BP1 proteins and longer-time persistence of γH2AX/53BP1 repair foci in cell nuclei have previously been associated with a radiosensitive phenotype of radiotherapy-experiencing patients, i.e., with compromised DSB repair due to suboptimal cooperation of repair factors [57]. This explanation can be reasonably acceptable in general but is hardly compatible with a radio-resistant character of U87 cells [50]. On the other hand, although the relevance to the results described here remains to be elucidated, Ochs et al. [41] revealed that silencing 53BP1 or altering its ability to bind damaged chromatin shifts limited DSB resection towards hyper-resection and, consequently, error-free gene conversion towards mutagenic single-strand annealing (SSA [58]). 53BP1 may thus foster the fidelity rather than final efficiency of DSB repair, which could indeed be expected in aggressive tumor cells. In accordance, although we observed more γH2AX/53BP1 repair foci in U87 cells at 24 h post-irradiation, the same holds true also for non-irradiated cells.

In both cell types, a considerable fraction of repair foci and clusters persisted in cell nuclei also after 24 h of repair. Since it has been shown that heavily damaged cell nuclei can maintain repair activity over several days [48,59], this observation may suggest that the cells are still actively processing complex damage introduced into DNA by relatively high doses (1.3 or 4 Gy) of high-LET (about 180 keV μm^−1^) ^15^N-radiation. Indeed, in accordance with some other reports (e.g., [58]), we have shown for other types of heavy ions, boron and neon (^11^B: LET = 138.1 keV μm^−1^, E = 8.1 MeV per n; ^20^Ne: LET = 132.1 keV μm^−1^, E = 46.6 MeV per n), that the average number of γH2AX/53BP1 foci per nucleus can (^11^B) or cannot (^20^Ne) return back to the baseline in irradiated (2 Gy) NHDF fibroblasts after a longer period post-irradiation (96 h PI) [1]. Alternatively, in accordance with what has been proposed in [1,60], such a long persistence of DSBs, together with their high complexity revealed, could indicate that the repair remains incomplete and the cells are going to die, unless they manage their survival by some other processes. A high radio-resistance of U87 cells could therefore also be ascribed to their adaptation for survival with unrepaired DNA. This may be a result of loss of function in some aspects of DNA repair which is supported by slower repair of glioma cells and the persistence of high levels of Rad51 and DNA-PK in U87 cells throughout the cell cycle [61].

Only cluster formation and relaxation were investigated in this study. Whether the clusters of 53BP1 (and other repair proteins) differ in their topology [38], for instance as a consequence of different damage complexity, structure or type of damaged chromatin (e.g., heterochromatin vs. euchromatin), and repair mechanism initiated (NHEJ, and HR), remains to be elucidated. This will be a subject of future investigations aimed at better understanding how the cluster topology and chromatin architecture contribute to the cell decision for a certain repair pathway at a given damaged chromatin site. Systematic investigations of spatial organization of γH2AX foci and sub-units, their topology in relation to chromatin environment followed by detailed structure measurements of repair protein arrangements at given repair loci may lead to a conclusive description on why certain repair proteins are recruited to a given damage site and how the repair pathway choice of a cell is determined. This may considerably impact the understanding of repair mechanisms and individual radio-sensitivity, as it is a matter of fact in radiation tumor treatment.

## 4. Materials and Methods

### 4.1. Cell Culture

**Figure 4:**
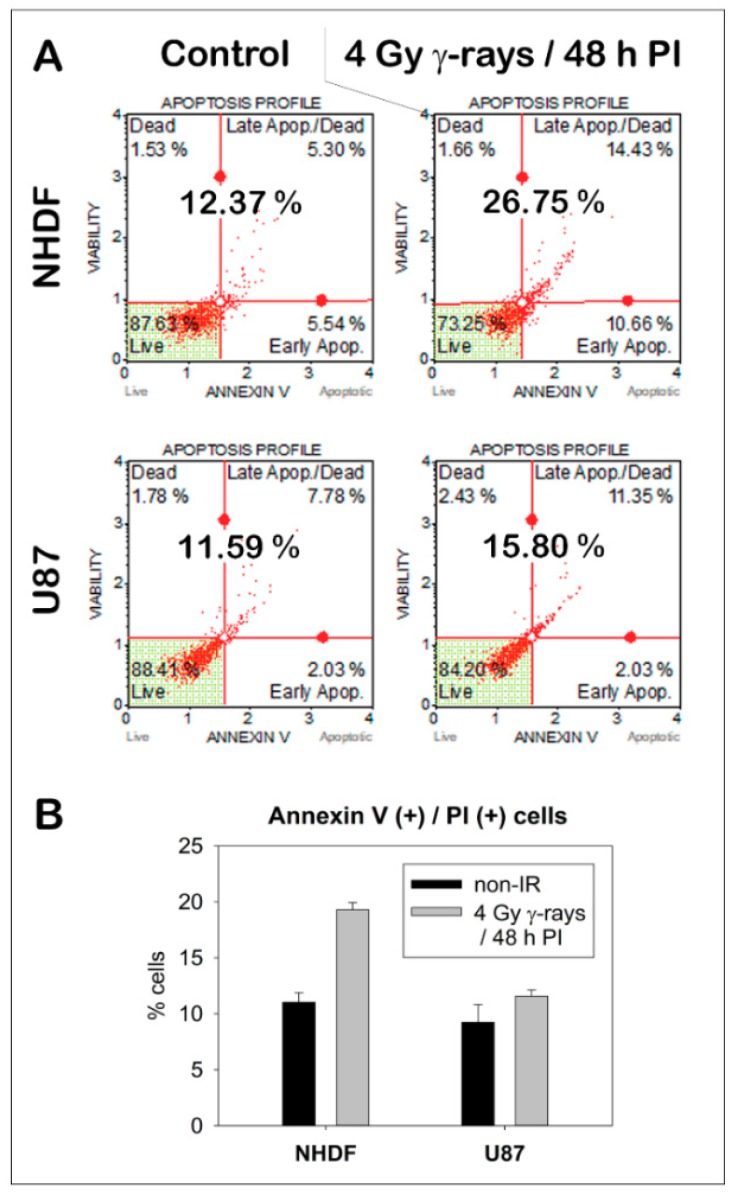
Cell death upon γ-irradiation compared for NHDF fibroblasts and U87 glioblastoma cells as quantified by flow cytometry 48 h post-irradiation (PI). (**A**) Illustrative flow-cytograms for NHDF and U87 cells prior to (left, control) and 48 h after irradiation with 4 Gy of γ-rays. x-axis: Annexin V positivity (a.u.); y-axis: propidium iodide (viability) positivity (a.u.); lower-left quarter (Annexin V (−)/Propidium Iodide (−)) = viable cells; lower right quarter (Annexin V (+)/Propidium Iodide (−)) = early apoptosis; upper left quarter (Annexin V (−)/Propidium Iodide (+)) = dead cells; upper right quarter (Annexin V (+)/Propidium Iodide (+)) = dead cells or late apoptosis shortly before dying. The total proportion of cells in the different quarters is given in percent of all cells measured. (**B**) Mean proportions (± standard deviation) of cells positive for Annexin V, Propidium Iodide (PI), or both these markers (white quarters in (**A**)) before (black columns) and 48 h after (grey columns) exposure of cells to 4 Gy of γ-rays.

As described in [40], primary neonatal human dermal fibroblasts (NHDF) and human U87 glioblastoma cells were cultured in Dulbecco’s modified Eagle medium (DMEM) supplemented with 10% fetal calf serum (FCS) and a 1% gentamicin–glutamine solution (all reagents from Sigma-Aldrich). These two cell types were used because of their high difference in radio-resistance which has been verified using the Annexin V/Propidium Iodide assay and quantification of relative cell death by flow cytometry before and after exposure to 4 Gy γ-irradiation (Figure 4).

For the ion-radiation experiments described in Section 2, the cells were maintained in T 25 cell flasks at 37 °C in a humidified atmosphere with 5% CO_2_. Sixteen to eighteen hours before radiation treatment, cells were seeded on the glass bottoms of Petri dishes and further cultivated until 80% confluence. For irradiation, the dishes were aseptically closed and sealed with Parafilm M (Sigma-Aldrich, Prague, Czech Republic). Irradiation took place at room temperature. After irradiation, the samples were further cultivated at 37 °C. At certain time points (5 min, 15 min only for 10°, 30 min, 45 min only for 10°, 1 h, 2 h, 4 h, 8 h, and 24 h) after irradiation aliquots of the same culture were taken and fixed with 4% formaldehyde/PBS (phosphate-buffered saline) for further labeling and SMLM analysis. This had the advantage that the cells used for the different repair periods and analyses were obtained from the same cell culture. This procedure was repeated independently with another cell culture.

### 4.2. Ion Irradiation

As recently described in [1], Nitrogen (^15^N) ions were accelerated (for details see Table 1) using a U 400 M isochronous cyclotron in the Flerov Laboratory of Nuclear Reaction at the Joint Institute for Nuclear Research (JINR, Dubna) [62]. Cells were irradiated on glass coverslips (on Petri dish bottoms) tangentially, i.e., with a 10° angle between the ion beam and the cell layer, or perpendicularly, i.e., with a 90° angle between the ion beam and the cell layer. The side of the coverslips covered with cells was oriented towards the ion beam so that the cells were hit by the particles before the beam continued into the culture medium in the Petri dish.

Non-synchronized cell populations with prevalent (>80%) G1 cells were irradiated in the culture medium with a dose of 1.3 Gy (tangential irradiation scheme; 10°) and 4 Gy (perpendicular irradiation scheme; 90°) at 37 °C. During irradiation, the cells were kept in a thermostable box, ensuring a constant temperature and prevention from infection during the whole procedure. After irradiation, the cells were immediately placed back into the incubator (37 °C/5% CO_2_) until fixation. The energy and corresponding LET values of ions in the plane of the cell monolayer were calculated using LISE++ software [63].

### 4.3. Immunofluorescence Staining

At the given time points (see Section 4.1), the cells were fixed in 4% formaldehyde/PBS (prepared freshly from paraformaldehyde) for 10 min and washed two times for 5 min in 1× PBS, permeabilized for 6 min in 0.2% Triton X-100 at RT, washed again three times for 5 min at room temperature (RT) in 1× PBS, and incubated in 2% bovine serum albumin (BSA) for 60 min at RT. Rabbit anti-53BP1 (ab21083, Abcam, Berlin, Germany) antibodies were diluted in the blocking solution I (1:600) and applied to the cells for 10 min at RT and subsequently overnight at 4 °C. The cells were then rinsed with 0.2% Triton X-100 and washed three times with 1× PBS for 5 min at RT. The secondary antibodies were AlexaFluor 594-conjugated goat anti-rabbit (Johnson Laboratories, New Brunswick, NJ, USA) antibodies. The antibodies were diluted in blocking solution II (1:400) and applied to the cells for 30 min (RT, in the dark). After incubation, the cells were washed three times in 1× PBS for 5 min. The cell nuclei were counter-stained with DAPI for 5 min at RT at a dilution of 1:20.000. After washing three times in 1× PBS for 5 min each, the cover slips were air dried, and the specimens were embedded in ProLong Gold^®^ (Thermo Fisher Scientific, Waltham, MA, USA), which was left to polymerize for 24 h in the dark at RT. Finally, the slides were sealed and stored in the dark at 4 °C.

### 4.4. Confocal Microscopy

Confocal microscopy images were acquired using an automated high-resolution Leica DM RXA microscope (Leica, Wetzlar, Germany) equipped with a Plan Fluotar oil-immersion objective (100×/NA1.3), a CSU 10a Nipkow disc (Yokogawa, Japan), a CoolSnap HQ CCD-camera (Photometrix, Tuscon, AZ, USA), and an Ar/Kr-laser (Innova 70C Spectrum, Coherent, Santa Clara, CA, USA). Automated image acquisition using Acquiarium software was described previously [1]. Forty serial optical sections were recorded at 0.25 μm step size along the z-axis.

### 4.5. Single Molecule Localization Microscopy (SMLM)

For data acquisition, the localization microscope from the light microscopy facility of the German Cancer Research Centre (DKFZ) was used. A detailed description of the instrument has recently been published elsewhere [29,34,51]. The microscope has an oil-objective (100×/NA 1.46) and four lasers: 405, 491, 561, and 642 nm with maximal laser powers of 120, 200, 200, and 140 mW, respectively. An in-built electron multiplier (EM-gain) enhances signals detected by the EmCCD camera (80 nm/px). To minimize drifts, the microscope was installed on a Smart-Table, compensating for vibrations, and provided with a water-cooling system, to keep constant temperature. Lasers were controlled using the “Omicron Control Center” program and image acquisition was carried out using “Live Acquisition v. 2.6.0.14” software.

Criteria for the selection of cells were size (not larger than a G2 nucleus), form (no twisted or broken nuclei taken) and visibility of the track (for 10°-irradiation). Searching was performed with minimal laser powers (4–12% maximum intensity). After an appropriate cell was found, a region of interest (ROI) was chipped to the size of the nucleus and the focal plane was adjusted to the repair foci or the track of repair.

A protocol for automatized localization image acquisition was used [45,51]. In brief, an initial excitation (3 s at maximum laser power) switches most fluorophores into the reversibly bleached state, and then 2000 single images are taken at maximum laser power and exposure of 100 ms per image. Measurements were done with the 561 nm wavelength laser. Wide-field images were always taken before localization image acquisition. For each time point, a minimum of 23 cells were imaged. The data stacks were stored as *.tif stacks and subjected to further computational analysis as described in Section 4.6.

### 4.6. Data Analysis for Single Molecule Localization Microscopy

Super-resolution signal coordinates were calculated using in-house Matlab-based software, as described in [44,45,51,64]. Background levels were multiplied by threshold factors (th = 4 for U87 and th = 5 for NHDF) for more rigorous background subtraction. Additionally, the first 500 frames of each data stack were discarded to further reduce background. Each remaining signal was then fitted with a 2D Gaussian curve. The intensity maximum determined the x-, y-coordinates of a signal point with a certain localization error which was about 11 nm for the measurements done here. The resulting localization data were saved in a data matrix containing the signal coordinates along with additional data (Table 2).

Density images were created, which mimic the visual impressions of conventional fluorescence microscopy: For each nucleus, the intensity values for each pixel (pixel size 10 × 10 nm^2^) were calculated according to the density of surrounding signals inside a radius of 1000 nm. The resulting intensities are normalized and blurred with a 50 nm sized Gaussian filter. The resulting image has a lower resolution than provided by the original data, but better visualizes the signal density and allows comparison with images obtained via conventional light microscopy.

For quantitative super-resolution data analyses, the total number of signal points and all-to-all point distances between signal points were calculated. Resulting single-cell data were summarized for each experimental setup for further statistical analysis of DNA damage repair dynamics and nano-spatial analysis of repair proteins.

**Figure 5:**
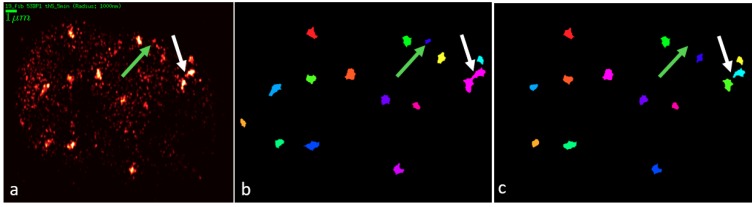
Exemplary images for a NHDF cell (90° irradiation) for cluster parameter comparison: (**a**) density-weighted image produced; (**b**) cluster image with minimum N = 63 neighbors; and (**c**) cluster image with minimum N = 84 neighbors. The white arrows show a separated cluster group in (**c**) and the green arrow a background signal wrongly counted as a cluster in (**b**). The different colors of the clusters are randomly chosen for better separation.

Cluster analysis was performed according to interactively determined parameters (Figure 5): Pixel size = 10 nm, radius = 20 pixels, maximum distance for all distances = 200 nm, and maximum distance for next neighbors = 200 nm. The minimal neighbor value (N) was determined for each cell line separately. For 90°-irradiated cells, the optimal neighbor values were N = 55 for U87 and N = 84 for NHDF cells. For the 10°-irradiated slides, N = 65 was chosen for both cell lines. Mean number of clusters per cell, number of signals in clusters and cluster areas were calculated.

## 5. Conclusions

A better understanding of the mechanisms behind repair protein foci formation and separation in sub-units (clustering) after complex DNA damaging through high-LET particle radiation could shed new light on nuclear organization of DNA repair processes and their activation upon specific conditions. In the present study, we revealed a cell type-dependent behavior of repair clusters as defined by SMLM parameters at the nanoscale. We assume that the differences in 53BP1 protein recruitment and dynamics of such clusters in formation and relaxation may be correlated to different radiation sensitivity of the cell types studied as well as different demands on 53BP1 by ongoing repair mechanisms. Technically, we show that SMLM opens door for new investigations providing much deeper (nanoscale) insights into the organization of repair foci and repair processes. By means of suitable procedures of quantitative analyses, the dynamics of functionally relevant sub-units of foci, i.e., appropriately defined clusters, could be validated also for ion-irradiation and hypotheses about long-time persistence were verified. Such ultra-resolution measurements of repair foci architectures offer new perspectives to comprehend the factors responsible for the cell’s decision for a particular repair pathway at a given damaged chromatin site, and, thus, repair efficiency and fidelity.

## Figures and Tables

**Table 1:** Irradiation Parameters for Different Irradiation Geometries.

Irradiation Angle	Energy (MeV/n)	LET (keV/µm)	Fluence (10^6^/cm) Per 1 Gy	Mean Number of Particles/Nucleus
10°	13.1	181.4	3.40	2.1
90°	13.0	182.9	3.41	25.4

**Table 2:** Structure of the Localization Data Matrix.

Column-Number	Type of Data
1	Signal-amplitude in photoelectrons
2	Lateral y-coordinates in nm
3	Lateral x-coordinates in nm
4	Lateral y-coordinates error in nm
5	Lateral x-coordinates error in nm
6	Standard-deviation σ_y_ in nm
7	Standard-deviation σ_x_ in nm
8	Number of photoelectrons in the signal
9	Number of the picture where the signal was detected
